# Electrospun Silk Fibroin–Silk Sericin Scaffolds Induced Macrophage Polarization and Vascularization for Volumetric Muscle Loss Injury

**DOI:** 10.3390/jfb16020056

**Published:** 2025-02-10

**Authors:** Yuqing Wang, Fangyu Ye, Xinbo Wei, Manman Wang, Zheng Xing, Haifeng Liu

**Affiliations:** 1 School of Integrated Chinese and Western Medicine, Anhui University of Chinese Medicine, Hefei 230012, China; zlxxwyq@163.com (Y.W.); yefangyubeijing@163.com (F.Y.); 2 Key Laboratory for Biomechanics and Mechanobiology (Beihang University) of Ministry of Education, Beijing Advanced Innovation Center for Biomedical Engineering, School of Biological Science and Medical Engineering, Beihang University, Beijing 100083, China; xinbowey@foxmail.com; 3 Key Laboratory of Xin’an Medicine, Ministry of Education, Anhui University of Chinese Medicine, Hefei 230038, China; mm-wang@ahtcm.edu.cn; 4 School of Pharmacy, Changzhou University, Changzhou 213164, China; xingzheng@cczu.edu.cn

**Keywords:** volumetric muscle loss, vascularization, macrophage polarization, silk fibroin, silk sericin

## Abstract

Volumetric muscle loss (VML) results in the impediment of skeletal muscle function. Tissue engineering scaffolds have been widely developed and used in skeletal muscle regeneration. However, scaffold implantation causes an immune response that endogenously regulates implant integration and tissue regeneration. Moreover, vascularization is thought to be a principal obstacle in the reconstruction of skeletal muscle defects. Thus, creating a pro-regenerative microenvironment that facilitates muscle regeneration and supports angiogenesis represents a promising strategy for tissue repair following volumetric muscle loss (VML) injury. Previously, the electrospun silk fibroin–silk sericin (SF-SS) film could regulate macrophage polarization and promote neovessel formation. This study aimed to investigate if the electrospun SF-SS scaffold was capable of supporting functional muscle regeneration. The results indicate that the conditioned medium collected from macrophages co-cultured with the 7:3 SF-SS scaffold significantly enhanced the proliferation and migration of myoblast C2C12 cells and improved the tube formation of HUVECs. Data from animal studies showed that the 7:3 SF-SS scaffold significantly enhanced M2 macrophage polarization, vascularization, and muscle fiber regeneration, reduced fibrosis, and improved muscle function after VML injury, thereby promoting the repair of muscle tissue. Therefore, the 7:3 SF-SS scaffold might represent a potential candidate for skeletal muscle regeneration following VML injury.

## 1. Introduction

Skeletal muscle generally possesses remarkable regenerative capability [1]. However, it faces challenges in the repair of volumetric muscle loss (VML) injuries, characterized by extensive damage exceeding 20% of the muscle weight [2]. Instead of activating endogenous self-renewal mechanisms, VML injuries often result in the deposition of excessive fibrous tissue at the site of the defect, leading to functional impairments. It has been reported that VML injury often arises from surgical procedures, denervation or high-energy trauma, such as crush injuries, traffic injuries, and falls from heights, contributing to the burden of disease worldwide [3]. Given the scarcity of autologous tissue donors, tissue engineering scaffolds have been developed as biomaterial substitutes to repair damaged muscles [4]. These scaffolds incorporate growth factors, bioactive molecules, or cells to facilitate tissue repair [4].

Skeletal muscle is renowned for its rich vascularity, which supports its high metabolic demands. The capillary networks within skeletal muscle not only provide sufficient oxygen and nutrients but also play a crucial role in regulating the myogenesis and remodeling process of skeletal muscle tissues [5]. Therefore, strategies to promote efficient revascularization are essential for muscle regeneration in VML injuries. Numerous approaches have been investigated to design vascularized muscle constructs capable of achieving the desired regenerative benefits [6,7]. For instance, scaffolds with specific structures or that release a growth factor, such as vascular endothelial growth factor (VEGF), can promote ingrowth of blood vessels into the defect site [8]. Jordana and colleagues highlighted this approach in their work, where VEGF-releasing scaffolds enhanced vascularization [8]. Similarly, Chen et al. engineered a hydrogel-based tissue construct with varying vascular network densities [9]. By manipulating hydrogel properties, they successfully facilitated accelerated muscle fiber repair through enhanced blood supply at the VML injury site [9].

Despite these advances, traditional tissue-engineering approaches for vascularization face several challenges. These include the reliance on an abundance of immune-compatible cells and their associated immune responses [10]. As a result, novel biomaterial-based strategies that harness in situ approaches to direct the immune response towards vascularization and tissue integration have emerged as promising alternatives [11,12]. For instance, Xiao et al. assessed the cellular and host responses of these 3D nanofiber scaffolds by comparing random membranes and mesh-like membranes with three different mesh sizes (250, 500, and 750 μm) [13]. The results showed that scaffolds with a mesh size of 500 μm were superior for M2 macrophage phenotype polarization, vascularization, and matrix deposition [13]. Moreover, while both poly (methacrylic acid–ethylene glycol) (MAA-PEG) and MAA-collagen hydrogels increased vascularization, the MAA-collagen hydrogel uniquely enhanced muscle innervation and significantly reduced the pro-inflammatory macrophage population (MHCII^+^CD206^−^), underscoring the critical role of macrophage modulation in tissue regeneration [14]. Macrophages are almost one of the first responses accompanied by a mass of bioactive production such as chemokines, cytokines, prostanoids, reactive oxygen species, and so on [15]. In terms of their function and surface markers, macrophages can be divided into a classically activated M1 phenotype and an alternatingly activated M2 phenotype [16,17]. Numerous studies showed that macrophage behavior can be influenced by designing or modifying biomaterials to promote angiogenesis by secreting pro-angiogenic cytokines or acting as support cells for vascular growth [18,19]. Further, Zhang et al. demonstrated that prevascularized porcine stomach musculofascial flap matrix enhanced neovascularization and constructive remodeling, marked by predominant infiltration of M2 macrophages and significant tissue formation in a VML model [20].

Silk is a natural composite fiber composed of silk fibroin (SF) and silk sericin (SS). SF is widely recognized for its excellent biocompatibility and minimal antigenicity, making it a well-established biomaterial [21]. SS, a protein coating the fibroin fibers, has historically been associated with the immunogenicity of native silk. However, extensive research has demonstrated that sericin elicits only mild inflammatory responses, exhibits negligible allergenicity, and shows low immunogenicity in vivo [22]. SS has also been an attractive biomaterial with great potential in tissue engineering and regenerative medicine [23,24]. In our previous work, we found that an electrospun film composed of a 7:3 ratio of silk fibroin (SF) to silk sericin (SS) exhibited the capability to activate a greater number of M2 macrophages, thereby driving angiogenesis [21]. Building on this finding, the objective of this study was to evaluate the therapeutic efficacy of SF-SS scaffolds in facilitating functional muscle regeneration in a VML model. We hypothesized that this immunomodulatory biomaterial could support angiogenesis and stimulate skeletal muscle regeneration by regulating macrophage polarization. In the study, we used conditioned medium collected from macrophages co-cultured with 10:0 and 7:3 SF-SS scaffolds to assess their capacity for proliferation and migration. Animal experiments were conducted to analyze the vascularization and muscle fiber regeneration implanted by 10:0 and 7:3 SF-SS scaffolds.

## 2. Materials and Methods

### 2.1. Preparation of SF and SS

Regenerated SF and SS were prepared following the method described in a previous study [21]. Bombyx mori cocoons were degummed in 0.02 M Na_2_CO_3_, dissolved in 9.3 M LiBr, dialyzed (3.5 kDa MWCO) against distilled water, and freeze-dried to obtain SF powder. The first degumming solution was collected, filtered, and subjected to the same dialysis and freeze-drying process as SF to obtain SS powder.

### 2.2. Preparation of Electrospun SF-SS Scaffolds

The SF sponge was dissolved in 1,1,1,3,3,3-hexafluoro-2-propanol (Aladdin, Shanghai, China) to obtain a uniform 10 wt.% SF solution, while the regenerated SS powder was dissolved in 200 μL of 98% formic acid. Electrospun SF-SS fibrous scaffolds with different mass ratios (10:0 and 7:3) were fabricated via electrospinning under controlled conditions: a flow rate of 0.2 mL/h, a voltage of 20 kV, and a needle-to-collector distance of 18 cm. The scaffolds were collected on a rotating collector to achieve a thickness of 3 mm and were subsequently cut into dimensions of 10 mm × 5 mm (length × width) for use in subsequent experiments.

### 2.3. Conditioned Media Collecting

The human monocytic cell line THP-1 (RRID: CVCL_0006) was purchased from the Cell Culture Center, Institute of Basic Medical Science, Chinese Academy of Medical Science, Beijing, China. The phorbol 12-myristate 13-acetate-differentiated THP-1 macrophages (5 × 10^5^ cells/mL) (PMA, P8139, Sigma-Aldrich, St. Louis, MO, USA) were trypsinized to seed on electrospun scaffolds within six-well culture plates for 3 days. Then, the conditioned media were collected and centrifuged at 300 g for 10 min to remove any residual, and stored at −70 °C for later use. Once used, they were mixed with the complete medium, which cells were cultured in subsequent experiments as 30%. The non-scaffold group was represented such that macrophages were directly seeded on six-well plate, and then the conditioned media were collected. The control group was a mixture of 1640 complete medium and the complete medium used in subsequent experiments.

### 2.4. Cells Culture

Mouse skeletal C2C12 myoblast cell line cells (RRID: CVCL_0188), purchased from the Chinese Academy of Medical Science, Beijing, China, were maintained in Dulbecco’s modified Eagle’s medium (DMEM, high glucose) supplemented with 1% penicillin/streptomycin (Solarbio, Beijing, China) and 10% fetal bovine serum (FBS, Corning, NY, USA) under a humidified incubator in 5% CO_2_ at 37 °C.

Human umbilical vein endothelial cells (HUVECs, RRID: CVCL_B7UI), obtained from the Chinese Academy of Medical Science, Beijing, China, were cultured in DMEM (high glucose) supplemented with 10% FBS and 1% penicillin/streptomycin. The cells were maintained in a humidified incubator at 37 °C with 5% CO_2_.

C2C12 cells were used to study muscle cell migration and differentiation, while HUVECs were used to model endothelial cell function and angiogenesis, providing insights into muscle and vascular biology.

### 2.5. CCK8 Assay

C2C12 cells were implanted into 96-well plates at the density of 4 × 10^4^ cells/mL for 24 h. Then the medium was replaced with the above conditioned medium, respectively, and incubated for 1, 3, and 5 days. The cells were replenished with the freshly prepared conditioned medium every 3 days. Lastly, the medium was discarded and 100 μL 1640 medium supplemented with 10% CCK8 (Beyotime Biotechnology, Shanghai, China) was added to each well for 1 h. The absorbance at a wavelength of 450 nm was evaluated using a microplate reader (Thermo Fisher Scientific, Waltham, MA, USA).

### 2.6. Wound Scratch Assay

C2C12 cells were seeded into 6-well plates at a density of 2 × 10^5^ cells/mL and allowed to grow into 90% confluence. Then, a scratch was gently induced with a sterilized pipette tip. The medium was discarded and washed with PBS twice. The cells were incubated with the freshly prepared conditioned medium in a 5% CO_2_ incubator at 37 °C. At the predetermined time (12 h), the cells were observed by microscope.

### 2.7. HUVEC Tube Formation

After thawing on ice, Matrigel (Corning, NY, USA) was plated in 96-well plates with 50 μL/well and allowed to polymerize for 45 min in a 5% CO_2_ incubator at 37 °C. HUVECs (6 × 10^5^ cells/mL) were then seeded onto the Matrigel with 30 μL per well, and conditioned medium was respectively combined with 70 μL and placed in a 5% CO_2_ incubator at 37 °C. The cells were observed under the microscope and imaged every 2 h to assess endothelial cell tubule formation. Total branch length, number of meshes, and number of junctions were quantified by ImageJ software (Version X; National Institutes of Health, Bethesda, MD, USA).

### 2.8. Implantation of Electrospun Scaffolds into Rat VML Injury Model

The animal protocols were reviewed and approved by the Animal Care and Use Committee of Anhui University of Chinese Medicine. The justification for the use of animals was that the animals were needed in research to evaluate the effect of electrospun scaffolds on muscle repair after VML injury. The animal model was used due to the fact that it approached a human context and avoided the risks associated with experimenting on humans. Twenty-four rats were housed in conditions of ventilation (15 to 20 air changes per hour), a relative humidity of 45–65%, and a temperature of 22 ± 2 °C. The rats were fed with food and water freely, and the cages and bedding were frequently changed. Measures were taken to minimize suffering, including the training of the operators, careful attention to the animals, gentle and quiet handling, and limitation of stressors imposed on the animals. To ensure the reliability and validity of the results, the principles of randomization and blinding in the experimental design were followed by the random number table method.

Sprague Dawley adult rats (male, 250–300 g) were bought from Henan Skobes Biotechnology Co., Ltd., Anyang, China. and allowed to acclimate for 2 weeks. The animals were first weighed, then administered an intraperitoneal injection of 1% sodium pentobarbital at 50 mg/kg for anesthesia. Over 20% of the tibialis anterior (TA) muscle was removed by a scalpel for establishing the VML injury model [2,25,26]. The defect dimensions were approximated 10 mm × 5 mm × 3 mm (length × width × depth). Disinfected electrospun 10:0 and 7:3 scaffolds were placed at the site of the defect. Single interrupted sutures were used to close the wound. After 1 week, 2 weeks, and 8 weeks, the TA muscle or implanted scaffolds were harvested from rats sacrificed for research. The group without any injury was used as the control. Rats were sacrificed by excessive anesthesia with 2% sodium pentobarbital intraperitoneally. There were at least three samples in every group at each time point.

### 2.9. Histology Evaluation

The harvested muscle tissues were preserved with 4% paraformaldehyde solution, dehydrated, embedded in paraffin, and sectioned at 5 μm thickness. Hematoxylin and eosin staining (H&E) and Masson’s trichrome staining (Servicebio, Wuhan, China) were performed on the sections. Immunofluorescent staining against different antibodies (collagen (COL I, GB114197-100), myosin heavy chain (MHC, GB112131-100), and CD31, GB300604) was also performed on the sections, respectively. All the antibodies were obtained from Servicebio. All in vitro staining was performed with triplicate samples per group. Three randomly selected fields of each section and three sections per specimen were observed with a light microscope (BX53, Olympus, Tokyo, Japan), and the digitized information was analyzed by ImageJ software.

### 2.10. Macrophage Polarization

For immunofluorescence analysis, monoclonal anti-CCR7 (ab32527) and anti-mannose receptor (CD206, ab8918) antibodies (1:200, Abcam, Cambridge, MA, USA) for rat macrophages were employed. Three randomly selected fields of each section and three sections per specimen were observed with a light microscope (BX53, Olympus, Tokyo, Japan), and image analysis was performed using IPP 6.0 to quantify both CCR7 and CD206 expressions at 7 and 14 days by means of the CCR7/CD206 IOD ratios.

### 2.11. Muscle Function Analysis

The TA muscle functional assessment of rats was conducted at 8 weeks after surgery [27]. The rats were anesthetized with 1% sodium pentobarbital intraperitoneally at 50 mg/kg and fixed. The TA was fully exposed and cut off at the end of the near toe. The one tendon was attached to the tension transducer (BL-420F, Chaoyang Instruments, Chengdu, China). The positive and negative electrodes were inserted into the muscle tissue, respectively. Ringer’s fluid was continually added to keep the muscle tissue moist. The effect of stimulation intensity on skeletal muscle contraction was first tested to obtain the optimal stimulation intensity with the following parameter settings: the intensity was 1 v, the wave width was 100 ms, and the intensity increment was 1 v. Then, under the optimal stimulation intensity, the peak isometric contractile force was recorded with different stimulation frequencies (from 1 Hz to 100 Hz).

### 2.12. Statistics

Data from more than three independent experiments were analyzed as means ± standard deviations. Statistical analysis was performed using GraphPad Prism 5.0 software with one-way analysis of variance (ANOVA) and Tukey–Kramer’s test. Statistical significance was considered at ** *p* < 0.01 and * *p* < 0.05.

## 3. Results

### 3.1. SEM Images

**Figure 1:**
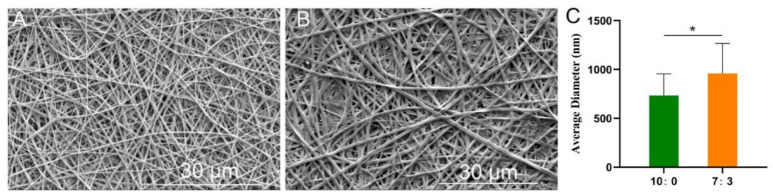
SEM images of scaffolds. (**A**) 10:0, (**B**) 7:3, (**C**) the average diameter, * *p* < 0.05, ($\bar{x}$ ± s, *n* = 6). Statistical analysis was estimated using Tukey–Kramer’s test.

As shown in Figure 1, the average fiber diameter of the 7:3 scaffold was larger than that of the 10:0 scaffold, consistent with our previous study [21]. The increase in fiber diameter with higher solution concentration can be attributed to the increased viscosity at higher concentrations. This higher viscosity reduces the elongation of the electrospinning jet, leading to the formation of thicker fibers [28].

### 3.2. Conditioned Media from Electrospun Scaffold-Treated Macrophages Drives C2C12 Cell Growth

**Figure 2:**
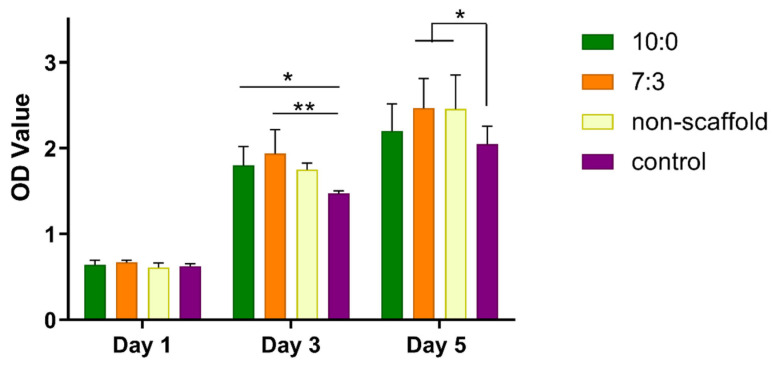
CCK-8 assays for C2C12 cell viability with the conditioned media, * *p* < 0.05, ** *p* < 0.01, ($\bar{x}$ ± s, *n* = 3). The non-scaffold group indicates the conditioned media collected from macrophages which were directly seeded onto six-well plates without scaffolds. The control group means the mixture of 1640 complete medium and the complete medium.

Macrophages were co-cultured on the electrospun scaffolds for 3 days. Then, the conditioned media were collected for culturing the myoblast C2C12 cells. As shown in Figure 2, there was no significant difference of OD value presented among the groups on day 1. On day 3, 10:0 and 7:3 groups both promoted C2C12 cell growth compared to the control group. The cell viability in the 7:3 group still increased on day 5. Therefore, the conditioned media collected from macrophages co-cultured with the 7:3 electrospun scaffold could greatly enhance the proliferation of myoblast C2C12 cells.

### 3.3. Conditioned Media from Electrospun Scaffols-Treated Macrophages Promote C2C12 Cell Migration

**Figure 3:**
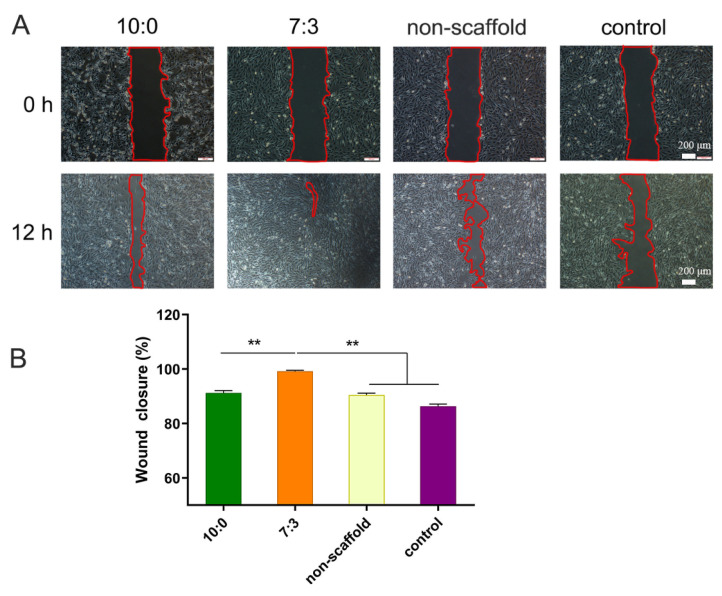
Scratch assay for C2C12 cell migration with the conditioned media. (**A**): represented images, (**B**): the percentage of wound closure. The statistical evaluation was performed with GraphPad Prism 9.0, ** *p* < 0.01 ($\bar{x}$ ± s, *n* = 3).

Next, we studied the effect of conditioned media on the migration of C2C12 cells. As shown in Figure 3, when C2C12 cells were cultured in different conditioned media for 12 h, the scratch widths of the 10:0 and 7:3 groups were reduced to a greater extent than those of the control and non-scaffold groups, and the scratch in the 7:3 group was almost full of cells and had the strongest migratory ability.

### 3.4. Tube Formation Assays

**Figure 4:**
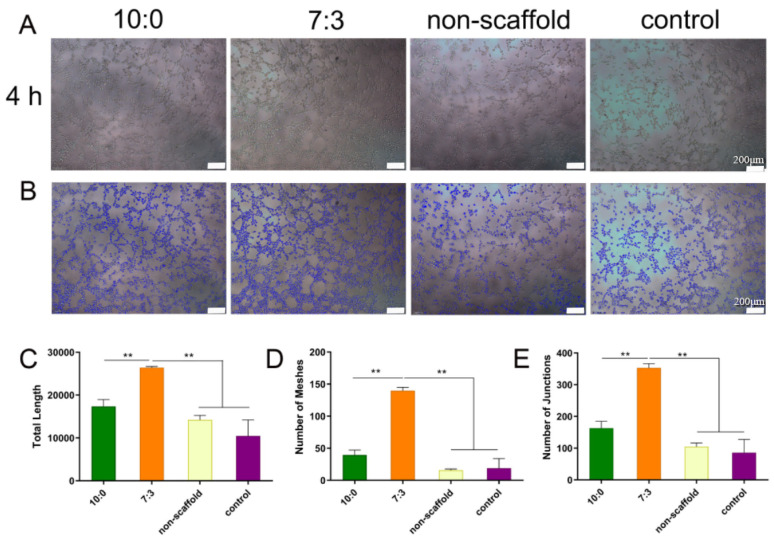
In vitro tube formation assay and quantification of HUVEC organization in different conditioned culture media. (**A**): photos of HUVEC cells cultured in different conditioned media for 4 h, (**B**): images processed with IPP 6.0 software, (**C**): total length, (**D**): number of meshes, (**E**): number of junctions. The statistical evaluation was performed with GraphPad Prism 9.0, ** *p* < 0.01 ($\bar{x}$ ± s, *n* = 3).

We also investigated the effect of the conditioned media on the tube formation of HUVECs. The results in Figure 4 showed that the 7:3 group was characterized by significantly higher total branch length, number of meshes, and number of junctions than other groups (** *p* < 0.01), which indicates that conditioned media from 7:3 electrospun scaffold-treated macrophages significantly enhanced tube formation in HUVECs.

### 3.5. Histology

**Figure 5:**
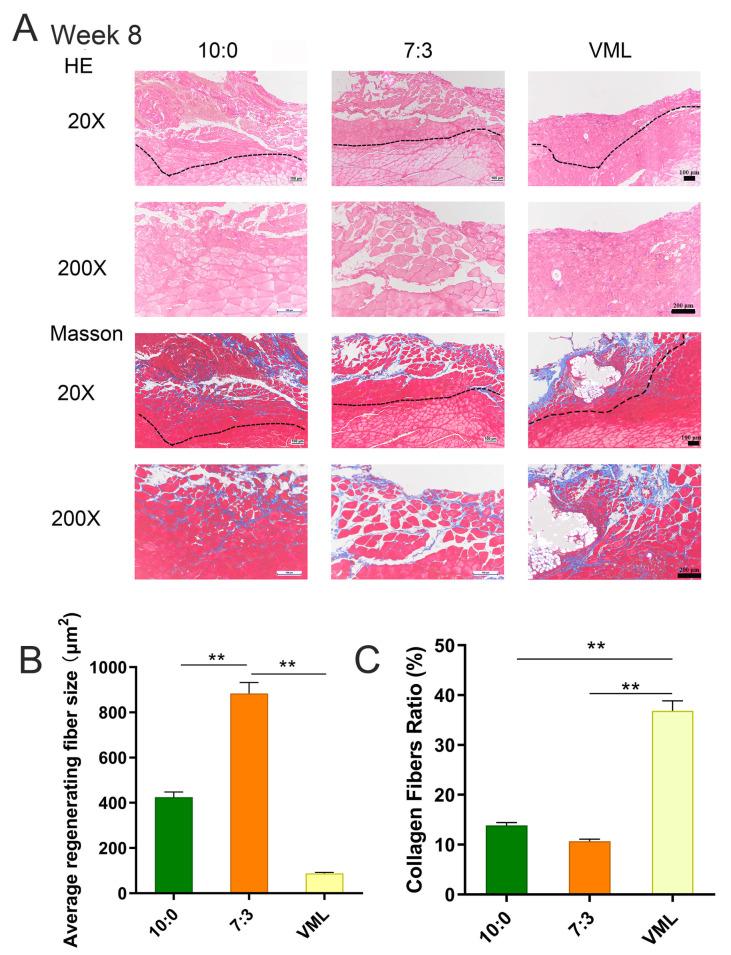
Morphology evaluation and quantified analysis of TA muscle at 8 weeks postinjury. (**A**): H&E staining and Masson’s trichrome staining of remodeled TA muscle in different groups at 8 weeks postinjury, (**B**): quantified analysis of average regenerating fiber size, (**C**): quantified analysis of surface ratio of collagen fiber deposition. Images were captured at 20× and 200× magnification. Scale bar, 100 μm. Black dotted lines in the images represent the boundary between the remaining muscle mass and the regenerated region. Statistical evaluation was performed with GraphPad Prism 9.0, ** *p* < 0.01 ($\bar{x}$ ± s, *n*= 3).

H&E staining as well as Masson’s Trichrome staining were conducted to assess the tissue morphology at the defect site. On day 7 and day 14, extensive cellular infiltration and muscle fibers were observed in the defect area in 10:0 and 7:3 groups (see Supplementary Materials Figures S1 and S2). These localized lesions of 10:0 and 7:3 groups showed an abundance of small, regenerated muscle fibers. The VML group showed less cell infiltration, fewer newly regenerating myofibers, and severe fibrosis. As shown in Figure 5, persistent collagen deposition was observed in all groups after 8 weeks. The 10:0 and 7:3 groups showed significant muscle recovery, such as increased nucleus location in the center of the muscle fibers. The regenerated muscle fibers were thinner and more disordered in the 10:0 group than those of the 7:3 group, although the 10:0 electrospun scaffold could induce regenerated muscle fiber. This finding indicates that 7:3 electrospun scaffold induced denser and more orderly regenerated muscle fibers with less collagen deposition.

### 3.6. Macrophage Polarization Toward M2 Phenotype

**Figure 6:**
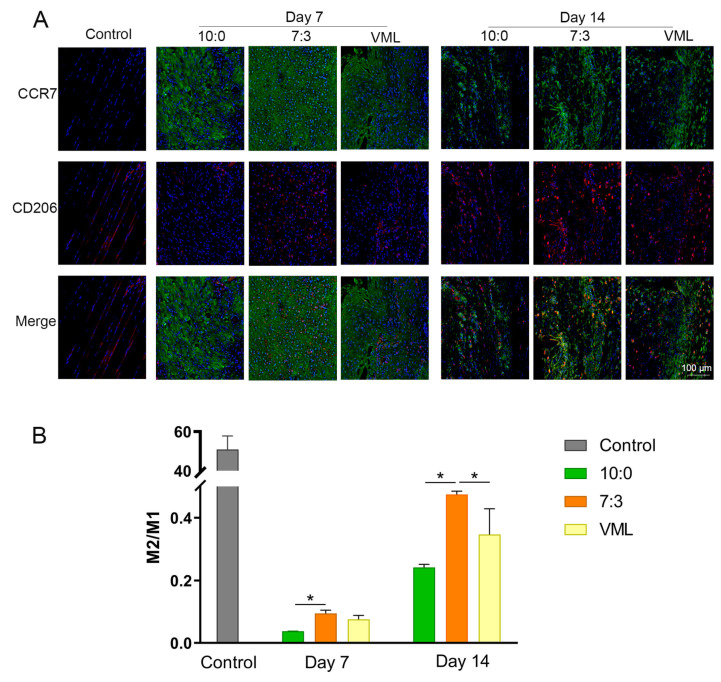
Macrophage polarization on days 7 and 14 postinjury. (**A**) Representative immunofluorescence images showing CCR7 (M1 marker, green) and CD206 (M2 marker, red). (**B**) Quantitative analysis of M2/M1 ratio performed using ImageJ. Statistical evaluation was conducted with GraphPad Prism 9.0, * *p* < 0.05 ($\bar{x}$ ± s, *n* = 3).

The polarization of macrophages was evaluated by immunofluorescence staining for CCR7+ (M1 macrophages) and CD206+ macrophages (M2 macrophages). In Figure 6, at 7 days postinjury, the groups overwhelmingly presented M1 macrophages. Until day 14, all groups exposed to injury demonstrated up-regulated M2 macrophages. The 7:3 group showed an increased CD206+ expression and a higher M2/M1 ratio compared to the 10:0 group and VML group.

### 3.7. Muscle Fiber Regeneration and Vascularization

**Figure 7:**
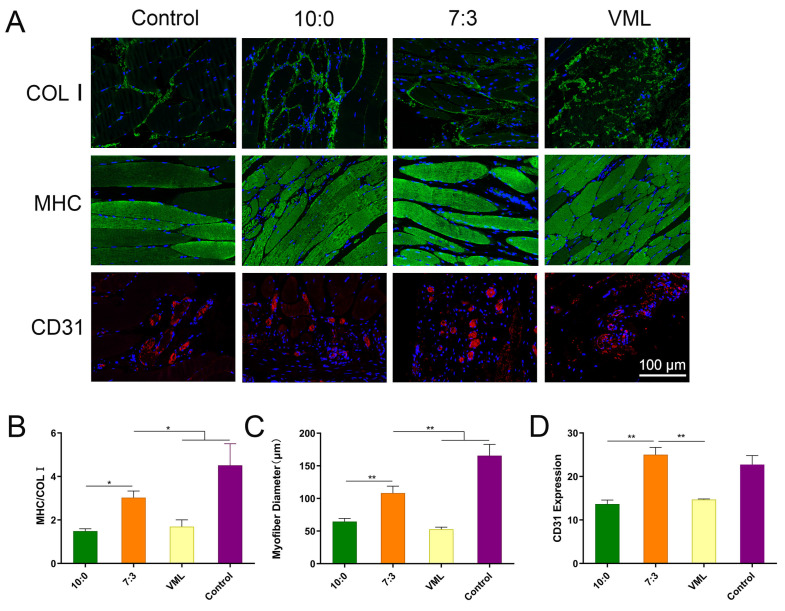
Muscle fiber regeneration. (**A**) Immunofluorescence staining of TA muscle remodeling in different groups 8 weeks postinjury, including COL I, MHC, CD31. Scale bar, 100 μm. (**B**) The ratio of MHC/COL I. (**C**) Myofiber diameter at 8 weeks postinjury. (**D**) CD31 expression. Statistical analysis was performed with GraphPad Prism 9, * *p* < 0.05 and ** *p* < 0.01 ($\bar{x}\;$ ± s, *n* = 3). Significance was estimated using ANOVA for multiple comparisons.

The extent of regeneration and fibrosis in the injured muscles was determined by immunostaining with MHC as well as COL I, and the new blood vessels were stained by CD31 (Figure 7). The fluorescence intensity ratio of MHC and COL I in the defect area was analyzed semi-quantitatively for the evaluation of regeneration and fibrosis (Figure 7) [29,30,31]. The muscles subjected to VML injury had significantly lower MHC/COL I ratios and significantly thinner regenerated muscle fibers compared with the control group (Figure 7A,B). Compared with the VML and 10:0 groups, the MHC/COL I of the 7:3 group was increased, and the regenerated muscle fibers were also significantly thickened. The muscle fibers in the 7:3 group were organized and more similar to those in the control group.

CD31 is a useful marker of vascular network maturity [32]. The expression level of CD31 in the 7:3 group was significantly higher than that in the 10:0 and VML groups, indicating that vascularization of remodeled muscles was gradually established (Figure 7A,D).

### 3.8. Functional Analysis

**Figure 8:**
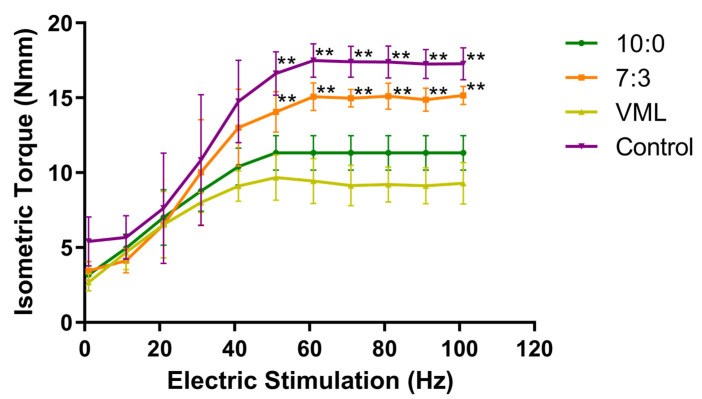
Functional evaluation of TA muscle exposed to VML injury ($\bar{x}$ ± s, *n* = 3), ** *p* < 0.01 vs. control.

We measured the functional recovery of TA muscle after 8 weeks postinjury. The results in Figure 8 showed that the muscle function in the VML group was significantly decreased compared with the control group (** *p* < 0.01) when the frequency of the electric stimulation was above 50 Hz. In addition, the 7:3 group could significantly improve the mechanical function of the injured muscle (** *p* < 0.01), which was still lower than the control group with the frequency of 50 Hz.

## 4. Discussion

In this study, we investigated the feasibility of electrospun scaffolds as candidates for skeletal muscle construct engineering. Morphological characterization of the electrospun scaffolds revealed that the 7:3 scaffold exhibited a larger average diameter than the 10:0 scaffold, with increased solution concentration leading to higher viscosity, which restricted jet elongation and produced thicker fibers. It is well known that the surface morphology of electrospun fibers, including fiber diameter and porosity, influences cell behaviors such as adhesion, proliferation, and infiltration. The effects of the morphology and physical attributes of the material itself on macrophage polarization should be the focus. Actually, it was the multiple variables, including sericin content, nanofiber diameter, and hydrophilicity, that affected the macrophage response. It was a result of comprehensive factors. A recent study indicated that the large-pore shish-kebab structure fiber scaffolds demonstrated superior immune performance, less macrophage aggregation, and easier differentiation to the anti-inflammatory M2 phenotype, which is known to be beneficial for tissue repair and regeneration [33]. This is a potential strategy for designing biomaterial scaffolds to adjust macrophage behavior for tissue regeneration. Herein, we investigated how different electrospun scaffolds influence macrophages in promoting tissue regeneration. Through in vitro experiments, we utilized C2C12 cells to study muscle cell migration and differentiation while concurrently modeling endothelial cell function and angiogenesis with HUVECs, thereby gaining insights into muscle and vascular biology. Furthermore, when combined with in vivo experiments, our findings revealed that 7:3 electrospun scaffolds induced regeneration, a process regulated by macrophages that secreted various cytokines with profound effects on endogenous cells.

The polarization of macrophages into distinct phenotypes, M1 and M2, plays a vital role in modulating inflammatory and regenerative responses within damaged tissues [34]. M1 macrophages are involved in proinflammatory processes that facilitate the migration and activation of myogenic progenitors [11]. On the other hand, M2 macrophages trigger a pro-regenerative response, stimulating the differentiation of muscle precursor cells and promoting myotube formation [35]. The sequential activation of M1 and M2 macrophages not only affects the outbreak and regression of inflammation but also has an important impact on tissue regeneration and remodeling [36]. Prolonged and uncontrolled inflammatory response (M1 macrophages) or inappropriate anti-inflammatory response can lead to poor tissue recovery [37,38]. Our data showed that a large number of M1 macrophages infiltrated into the defect site of the VML group on day 7 after injury, indicating that the defect site was still in an acute inflammatory microenvironment. The polarization of macrophage phenotype into M2 is advantageous for the repair of muscle tissue [25]. Therefore, in the rat model of VML injury, the M2/M1 ratio in the 7:3 group was higher than that in the 10:0 group and the VML group on days 7 and 14, representing a favorable condition for muscle tissue regeneration. Prior results of the subcutaneous tissue of healthy rats showed that the M2/M1 ratio in the 7:3 group was higher than that in the 10:0 group on day 7, while the M2/M1 ratio was lower than that in the 10:0 group on day 14, which might be due to the difference between the health status and pathological status of rats or the difference between skin and muscle tissue [39,40]. Remarkably, the 7:3 SF-SS scaffolds consistently up-regulated the fluorescence intensity of macrophage markers CCR7 (M1) and CD206 (M2) in both subcutaneous and muscle tissue, underscoring their capacity to induce macrophage activation.

Interleukin-10 (IL-10) is regulated by the inflammatory response and myogenesis and is a beneficial cytokine for muscle regeneration through immune regulation [41]. Our previous data showed that 7:3 scaffolds effectively activated macrophages, leading to higher secretion levels of IL-10 [21]. Notably, Deng et al. found that IL-10 plays an important regulatory role in the phenotypic transition of macrophages from M1 to M2 in injured muscle tissue in vivo, which is required for muscle growth and regeneration [42]. Mahon et al. revealed that the conditioned medium of macrophages treated with nanoparticles promoted the osteogenic effect of mesenchymal stem cells to restore bone defects in an IL-10-dependent manner, while exogenous IL-10 alone did not produce the same effect [43]. These results further explained the effects of 7:3 electrospun scaffolds treated by macrophages on myoblasts, C2C12 cells, and endothelial cells, and provided more clues to the mechanism of 7:3 electrospun scaffolds promoting repair of muscle exposed to VML injury. Additionally, further experiments such as PCNA and TUNEL staining using the differentiated C2C12 cells still need to be performed for skeletal muscle cell proliferation and apoptosis in future studies [44,45].

To further evaluate the effects of electrospun scaffolds on myofiber regeneration, the MHC/COL I ratio was used to quantify the degree of myofiber regeneration versus fibrosis [26]. At 8 weeks after injury, the MHC/COL I ratio was significantly increased in the 7:3 group, which indicates increased myofiber regeneration and reduced fibrosis. The 7:3 electrospun scaffolds significantly improved the muscle contractile function after VML injury but were still lower than the control group. The reason may be that VML injury is a chronic injury with a repetitive decay–regeneration cycle, and an increase in peak isothermal force may not be observed until 2–4 months after injury [46]. It is also hypothesized that regenerated muscle fibers (MHC+) do not recover innervation at week 8 and therefore do not produce sufficient contractile capacity [26]. Thus, even though vascular reconstruction plays an important supporting role, other factors will determine the outcome of muscle regeneration after VML injury [47].

## 5. Conclusions

In this study, the conditioned medium of macrophages co-cultured with 7:3 electrospun scaffolds could significantly promote the proliferation and migration of myoblast C2C12 cells and improve the tube-forming of HUVECs. The in vivo experiments showed that the 7:3 group significantly improved macrophage polarization to the M2 type, vascularization, and muscle fiber regeneration, and reduced fibrosis while improving muscle contractility and promoting muscle tissue repair after VML injury.

## Data Availability

The original contributions presented in the study are included in the article/Supplementary Materials. Further inquiries can be directed to the corresponding author.

## Supplementary Materials

The following supporting information can be downloaded at: https://www.mdpi.com/article/10.3390/jfb16020056/s1, Figure S1: Morphology evaluation of TA muscle at 7 days postinjury. Figure S2: Morphology evaluation of TA muscle at 14 days postinjury.
