# Supplementary material for: Electrospun Silk Fibroin–Silk Sericin Scaffolds Induced Macrophage Polarization and Vascularization for Volumetric Muscle Loss Injury
Source: J Funct Biomater. 2025 Feb 10;16(2):56. doi: 10.3390/jfb16020056 (PMC11856479; doi:10.3390/jfb16020056)
Supplement: Supplementary file 1 [file jfb-16-00056-s001.zip › jfb-3447815-supplementary.pdf]

Journal of
Functional Biomaterials

MDPI

Article

Electrospun Silk Fibroin–Silk Sericin Scaffolds Induced Macrophage Polarization and Vascularization for Volumetric Muscle Loss Injury

Yuqing Wang [1], Fangyu Ye [1], Xinbo Wei [2], Manman Wang [3], Zheng Xing [4] and Haifeng Liu [2,*]

[1] School of Integrated Chinese and Western Medicine, Anhui University of Chinese Medicine, Hefei 230012, China; zlxxwyq@163.com (Y.W.); yefangyubeijing@163.com (F.Y.)
[2] Key Laboratory for Biomechanics and Mechanobiology (Beihang University) of Ministry of Education, Beijing Advanced Innovation Center for Biomedical Engineering, School of Biological Science and Medical Engineering, Beihang University, Beijing 100083, China; xinbowey@foxmail.com
[3] Key Laboratory of Xin'an Medicine, Ministry of Education, Anhui University of Chinese Medicine, Hefei 230038, China; mm-wang@ahtcm.edu.cn
[4] School of Pharmacy, Changzhou University, Changzhou 213164, China; xingzheng@cczu.edu.cn
* Correspondence: haifengliu@buaa.edu.cn

Academic Editor: Sorina Dinescu

Received: 11 January 2025
Revised: 26 January 2025
Accepted: 8 February 2025
Published: 10 February 2025

Citation: Wang, Y.; Ye, F.; Wei, X.; Wang, M.; Xing, Z.; Liu, H. Electrospun Silk Fibroin–Silk Sericin Scaffolds Induced Macrophage Polarization and Vascularization for Volumetric Muscle Loss Injury. *J. Funct. Biomater.* **2025**, *16*, 56. https://doi.org/10.3390/jfb16020056

Copyright: © 2025 by the authors. Submitted for possible open access publication under the terms and conditions of the Creative Commons Attribution (CC BY) license (https://creativecommons.org/licenses/by/4.0/).

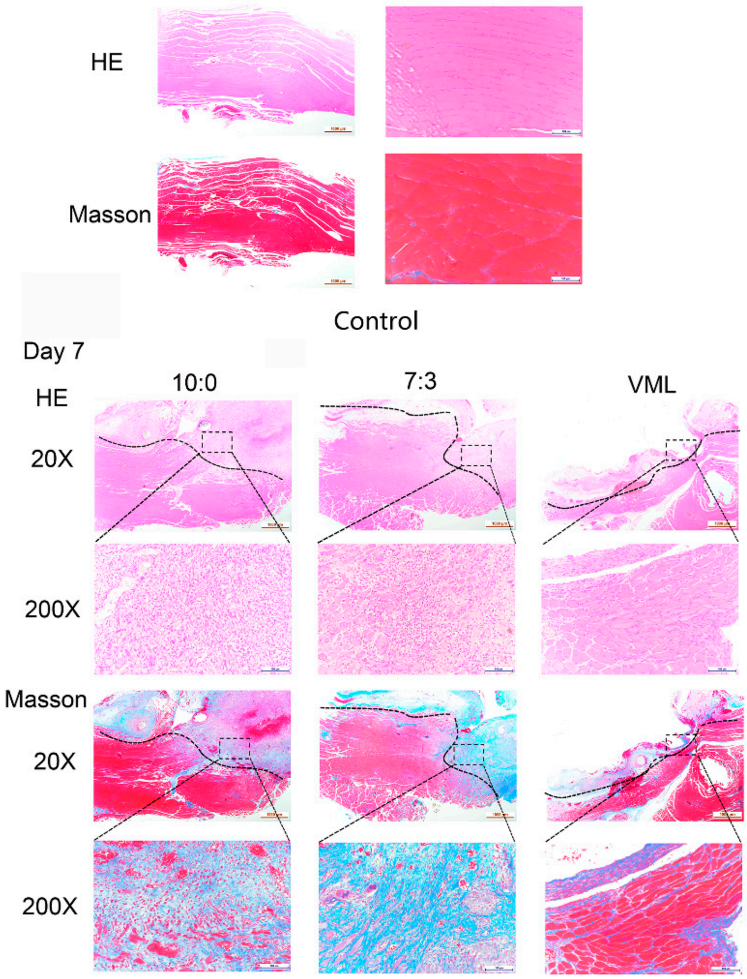

Figure S1. Morphology evaluation of TA muscle at 7 days postinjury. The images were taken at 4× magnification and 10× magnification. Scale bar, 1000 μm and 100 μm. The black dotted lines in the images indicated the boundary between the remaining muscle mass and the regenerate region.

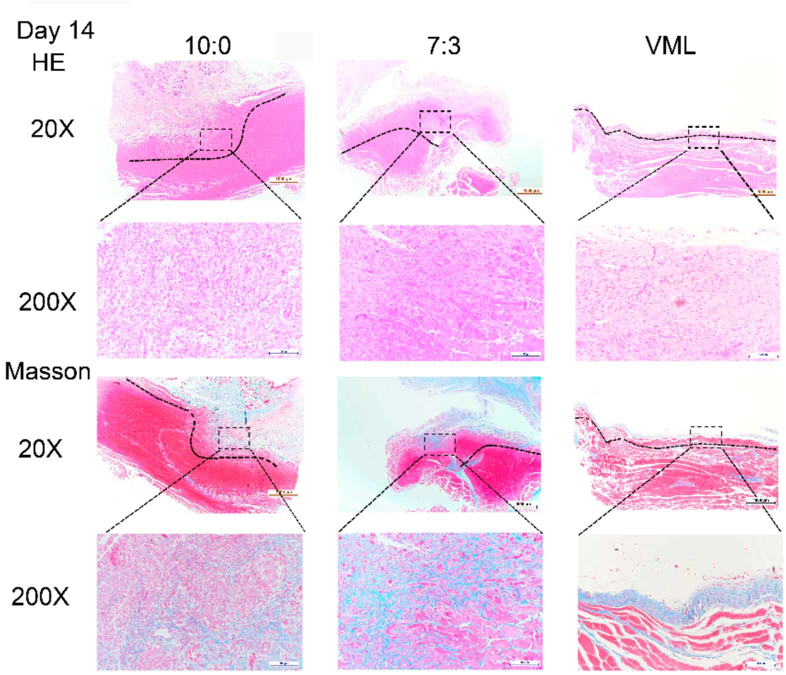

Figure S2. Morphology evaluation of TA muscle at 14 days postinjury. The images were taken at 4× magnification and 10× magnification. Scale bar, 1000 μm and 100 μm. The black dotted lines in the images indicated the boundary between the remaining muscle mass and the regenerate region.
